# Learning from lived experiences to address public health concerns in incarceration and community corrections

**DOI:** 10.1186/s44263-025-00192-4

**Published:** 2025-08-20

**Authors:** Gerrit John-Schuster

**Affiliations:** Springer Nature, BioMed Central, New York, NY USA

*Our collection on addressing public health concerns in incarceration and community corrections showcases varied lived experiences and health needs of people involved with the criminal justice system. We hope these can inform policy and decision-making in these settings.*

Incarceration poses a major public health challenge, as people who are or were incarcerated face elevated rates of chronic illnesses, infectious diseases, mental health issues, and substance use disorders (SUD). The prison environment often worsens these health problems or leads to new ones. In our collection guest-edited by Dr. Ingrid Binswanger, from Kaiser Permanente Colorado, USA, and Dr. Charlie Brooker, from Royal Holloway, University of London, UK, we called for research and opinion that explore the various health needs of people involved with the criminal justice system, such as in police stations, courts, jails, prisons, or probation/community corrections services, and that propose new approaches to addressing these issues (https://link.springer.com/collections/fdhhdecgja).

Most of the content that we published within this collection showcases lived experiences of people affected by SUD and mental illnesses. Strong-Jones et al. explored how incarceration and reentry negatively affected substance use outcomes in women with opioid use disorder (OUD) in Pennsylvania, USA [1]. The study found that women with OUD often experienced trauma during incarceration due to inadequate detoxification support and limited treatment options, compounded by stigma. Upon reentry, challenges such as unstable housing, unemployment, and returning to unsupportive environments increased relapse risk. Taylor et al. investigated a similar situation among legal-involved veterans in the USA, namely their access to medications for OUD (MOUD) within the Veterans Health Administration system [2]. The main findings were that Veterans Treatment Court policies both supported and restricted MOUD access, and that policies and preferences within the criminal justice system both enhanced and limited MOUD availability in jails and prisons. These studies highlight major inconsistencies in policies and approaches that affect treatment access and outcomes.

As people with histories of criminalized drug use face high risks of overdose, suicide, and infectious diseases after release from incarceration, there is a need to understand risk pathways for these outcomes. The review by Paquette et al. proposed a conceptual model outlining how post-release psychosocial needs, including basic, relational, medical, and mental health and substance use-related needs, contribute to these risks [3]. Vulnerabilities related to criminal legal involvement and substance use affect individuals at personal, interpersonal, institutional, community, and policy levels, hindering their ability to meet these needs. In turn, this might lead to situations of overwhelming competing demands after release, with negative emotions and risky behaviors, including continued drug use, disengagement with care, and self-harm. Ultimately, this could increase the risk for overdose, suicide, and infectious diseases.

Mental health in carceral settings is a global public health issue, with legal-involved people disproportionately affected by mental illnesses [4]. While many community supervision agencies for probation and parole have adopted innovative, evidence-based approaches to support this population, success in one region may not translate to others due to contextual differences like governance, culture, and resources. Implementation science (IS) research, defined as “the scientific study of methods to promote the systematic uptake of research findings and other evidence-based practices into routine practice, and hence to improve the quality and effectiveness of health services” [5], could assist in adapting and applying health interventions within criminal legal system settings, as outlined in a perspective by Van Deinse et al. [6]. Using specialized mental health community supervision in the USA as an example, the authors applied the EPIS framework (Exploration, Preparation, Implementation, Sustainment) to outline key steps and questions agencies should consider to successfully move interventions from theory into practice across varying contexts. In another study, Van Deinse et al. made use of IS research to improve a statewide specialized mental health supervision program in the USA [7]. Through rapid qualitative assessments in the pre-implementation phase, they were able to obtain important actionable insights that can significantly improve the effectiveness and efficiency of implementing such health interventions in real-world criminal justice settings.

As these articles and the lived experiences shared within them indicate, the health needs of people who are or were incarcerated are multi-faceted, with many factors and stakeholders contributing to long-term negative effects on health outcomes. In order to mitigate these issues, a collaborative effort is required involving those affected by incarceration to inform policy and design of novel health interventions.

## Data Availability

Not applicable.
